# Platelet-based biomarkers: A new frontier in inflammation monitoring for respiratory infections

**DOI:** 10.12688/f1000research.161124.1

**Published:** 2025-03-25

**Authors:** Seemitr Verma, Ruchee Khana, Vinay Khanna, Saisreeram Kandula, Vikram Ram Rajgopal, Vishnu Prasad

**Affiliations:** 1 Dept. of Pathology, Kasturba Medical College, Manipal, Manipal Academy of Higher Education, Manipal, Karnataka, India; 2 Dept. of Microbiology, Kasturba Medical College, Manipal, Manipal Academy of Higher Education, Manipal, Karnataka, India

**Keywords:** Platelet indices, Platelet Distribution Width, Erythrocyte sedimentation rate, C-Reactive Protein, Pulmonary Tuberculosis, Pneumonia

## Abstract

**Background:**

Platelets play a crucial role in haemostasis and have emerging functions in inflammation, particularly in respiratory infections like pulmonary tuberculosis (PTB) and pneumonia. The objective of this study was to investigate alterations in platelet parameters and their relationship with erythrocyte sedimentation rate (ESR) in PTB and pneumonia patients, comparing them to healthy controls, to evaluate their diagnostic and prognostic potential.

**Methods:**

This retrospective case-control study was conducted at a tertiary care center, involving 450 participants: 158 PTB patients, 142 pneumonia patients, and 150 healthy controls. Laboratory data including platelet count, mean platelet volume (MPV), plateletcrit (PCT), platelet distribution width (PDW), and ESR were obtained from the hospital’s Laboratory Information System. Statistical analyses, including Receiver Operating Characteristic (ROC) curve analysis, were performed to determine the diagnostic efficacy of the tests.

**Results:**

PTB and pneumonia patients exhibited significantly higher platelet counts and PCT, along with lower MPV compared to controls (p<0.001). In PTB patients, platelet count showed a positive correlation with PCT and ESR, and a negative correlation with MPV and PDW (p<0.001). Similar patterns were noted in pneumonia patients. ROC curve analysis identified cutoff values for platelet count (312,500/μl) and PCT (0.21%), which showed moderate sensitivity and specificity for differentiating PTB from pneumonia. ESR with a cutoff of 66.5 mm/h demonstrated comparable diagnostic performance.

**Conclusions:**

Altered platelet counts and indices in PTB and pneumonia patients correlate significantly with ESR levels, suggesting their potential as non-invasive diagnostic and prognostic markers in respiratory infections. Further prospective studies are needed to validate these findings and integrate platelet indices into clinical diagnostics

## Introduction

Platelets are best known for their role in preventing bleeding through hemostasis. However, recent research has shown their significant involvement in inflammatory processes, another critical biological function which are common in various infections. Beyond clot formation, platelets activate complement factors, facilitate chemotaxis—the movement of cells toward chemical signals—and indirectly support the immune system’s ability to engulf and destroy foreign invaders. ^1, 2^ Inflammatory responses are commonly assessed using indicators such as erythrocyte sedimentation rate (ESR) and C-reactive protein (CRP). ESR measures the rate at which red blood cells settle in a tube over one hour, serving as an indirect marker of inflammation due to alterations in plasma proteins. ^3^ Platelet activity can be quantified through specific parameters collectively known as platelet indices, including Mean Platelet Volume (MPV), Plateletcrit (Pct), and Platelet Distribution Width (PDW). MPV reflects the average size of platelets, Pct indicates the proportion of blood volume occupied by platelets, and PDW measures the variation in platelet size. ^4^

In conditions such as tuberculosis (TB), changes in platelet counts and indices, along with elevated acute phase reactants like ESR and CRP, have been documented. ^5– 9^ Reactive thrombocytosis, an increase in platelet count, has been reported in pulmonary tuberculosis, potentially driven by inflammatory interleukins such as IL-1 and IL-6 stimulating bone marrow platelet production. ^10– 13^ Additionally, studies have shown mixed results regarding MPV in inflammatory disorders, with some indicating higher MPV and others lower MPV during inflammation. ^14– 17^

Understanding the role platelets in inflammation is crucial for developing diagnostic and prognostic tools for various infections. Platelet indices offer a non-invasive means to assess the inflammatory status of patients, potentially aiding in the differentiation between conditions with similar clinical presentations, such as PTB and pneumonia. ^18, 19^

This study aims to explore the relationship between platelet indices and ESR in patients with respect to respiratory infections such as pulmonary tuberculosis and pneumonia, assessing whether these parameters can aid in diagnosing and differentiating between other inflammatory markers such as ESR and CRP. This research seeks to find the answer in the understanding of platelet dynamics in inflammatory responses associated with respiratory infections.

## Methods

### Objective

This study aimed to explore the changes in platelets, platelet-related indexes, and acute phase reactants in patients with pulmonary tuberculosis (PTB) and pneumonia, compared to healthy individuals. Specifically, the study sought to understand how platelets, beyond their well-known role in blood clotting, influence inflammatory processes. By comparing these parameters across the three groups and with other inflammatory markers, we aimed to determine whether platelets could serve as a better indicator of inflammation in pulmonary TB and pneumonia.

### Study design

This retrospective case-control study was conducted at a multispecialty tertiary care center with a capacity of 2500 beds. The study population consisted of 450 participants: 158 patients with clinically confirmed pulmonary tuberculosis, 142 patients diagnosed with pneumonia, and 150 healthy controls.

**Inclusion criteria:** Adult patients diagnosed with pulmonary tuberculosis (PTB) or pneumonia who provided informed consent.

**Exclusion criteria:** Patients under 18 years of age and those who refused consent to participate in the study.

### Data collection

Laboratory data, including platelet counts, mean platelet volume (MPV), plateletcrit (PCT), platelet distribution width (PDW), and erythrocyte sedimentation rate (ESR), were extracted from the hospital’s Laboratory Information System. Demographic information was obtained from medical records.

### Methodological details

**Pulmonary tuberculosis diagnosis:** Confirmed through clinical evaluation, radiological findings, and microbiological tests such as sputum smear microscopy and culture (reference to guidelines or specific criteria could be added for clarity).

**Pneumonia diagnosis:** Based on clinical signs, radiological imaging, and microbiological confirmation according to standard guidelines (specific guidelines or diagnostic criteria could be referenced for reproducibility).

### Statistical analysis

Data were analyzed using Microsoft excel 2010. Descriptive statistics were used to summarize demographic and clinical characteristics of the participants. Comparisons between groups were made using the following statistical tests:

ANOVA for continuous variables (e.g., platelet count, MPV, ESR).

Chi-square tests for categorical variables (e.g., gender, diagnosis).

Receiver Operating Characteristic (ROC) curve analysis was performed to evaluate the diagnostic performance of platelet count, PCT, and ESR in differentiating PTB, pneumonia, and healthy controls. The area under the curve (AUC) was used to assess diagnostic efficacy.

### Ethical considerations

Ethical approval was obtained from the institutional ethics committee (IEC no. 673/2019). All patient data were anonymized and confidentiality was maintained in accordance with ethical guidelines.

## Results

The platelet count of the Tuberculosis and Pneumonia groups were significantly higher than that of the Control group (p<0.001). MPV of the Tuberculosis and Pneumonia groups were significantly lower than the Control group (p<0.001). Platelet count, Pct and ESR of the Tuberculosis group were significantly higher than those of Pneumonia group (p<0.05). In the Tuberculosis group, Platelet count showed a negative correlation with MPV (r=-0.444, p=<0.001) and PDW (r=-0.412, p<0.001); and a positive correlation with Pct (r=0.831, p<0.001) and ESR (r=0.364, p<0.001). MPV also showed a positive correlation with PDW (r=0.675, p<0.001) and a negative correlation with Pct (r=-0.176, p=0.05). There was a negative correlation between PDW and Pct (r=-0.255, p<0.01). ESR positively correlated with Pct (r=0.296, p<0.001).

Similar to the findings in the Tuberculosis group, Platelet count negatively correlated with MPV (r=-0.501, p=<0.001) and PDW (r=-0.619, p<0.001); and positively correlated with Pct (r=0.774, p<0.001) and ESR (r=0.311, p<0.001) in the Pneumonia group as well. MPV showed a positive correlation with PDW (r=0.625, p<0.001) and a negative correlation with Pct (r=-0.399, p=0.001). PDW and Pct negatively correlated with each other (r=-0.661, p<0.01). ESR showed a positive correlation with Pct (r=0.196, p=0.019). The laboratory outcomes of platelet indices of each group are given in Table 1.

**Table 1.** Laboratory Outcomes of the TB group, Pneumonia group and Control group.

Variables	Tuberculosis (Group 1) (n=158)		Pneumonia (Group 2) (n=142)		Control (Group 3) (n=150)		Control-TB	Control-Pneumonia	TB and Pneumonia
	Mean	SD	Mean	SD	Mean	SD	P-value	P-value	P-value
Age (years)	50.1	16.4	61.3	14.5	48.6	11.1	0.358	<0.001 *	<0.001 *
PLT(x10 ^3^/μl)	374.6	147	323.2	130.9	247.5	53.7	<0.001 *	<0.001 *	0.002 *
MPV (fL)	7.6	1.1	7.7	0.9	8.4	0.7	<0.001 *	<0.001 *	0.428
PDW (%)	16.69	0.692	16.83	0.767	16.706	1.401	0.952	0.35	0.125
Pct (%)	0.257	0.1	0.206	0.088	0.313	1.305	0.597	0.327	<0.001 *
ESR (mm/h)	74.3	29.1	64.8	30.9	.	.			0.008 *

PLT: Platelet count; MPV: Mean Platelet Volume; PDW: Platelet Distribution Width; Pct: Plateletcrit; ESR: Erythrocyte Sedimentation Rate.

* Significant value (p < 0.05).

The two groups were further studied based on platelet count. Patients of each group were divided into two subgroups. In both these groups, MPV was significantly lower in patients with thrombocytosis compared to those having normal platelet count (p<0.001), whereas Pct and ESR were significantly higher in patients with thrombocytosis (p<0.001). PDW was significantly lower in patients with thrombocytosis (p<0.05). The platelet indices of the two subgroups of the Tuberculosis group are given in Table 2, and those of the Pneumonia group are given in Table 3.

**Table 2.** Platelet Indices and ESR of TB patients with normal Platelet count and those with Thrombocytosis.

Variables	Platelet Category		P-value
	150-400 (x10 ^3^/μl) (n=84)	>400 (x10 ^3^/μl) (n=61)	
Age (years)	49.6 (16.6)	49.9 (16.6)	0.89
MPV (fL)	7.8 (1.1)	7.2 (0.8)	<0.001 *
PDW (%)	16.8 (0.6)	16.5 (0.7)	0.02 *
Pct (%)	0.21 (0.05)	0.34 (0.09)	<0.001 *
ESR (mm/h)	67.5 (29.6)	84.3 (25.4)	<0.001 *

* Significant Value (p<0.05).

**Table 3.** Platelet Indices and ESR of Pneumonia patients with normal Platelet count and those with Thrombocytosis.

Variables	Platelet Category		P-value
	150-400 (x10 ^3^/μl) (n=95)	>400 (x10 ^3^/μl) (n=34)	
Age (years)	62.11 (14.68)	59.97 (14.43)	0.47
MPV (fL)	7.75 (0.83)	7.13 (0.73)	<0.001 *
PDW (%)	16.85 (0.64)	16.29 (0.39)	<0.001 *
Pct (%)	0.19 (0.06)	0.29 (0.87)	<0.001 *
ESR (mm/h)	60.45 (29.33)	78.67 (29.29)	<0.001 *

MPV, Mean Platelet Volume; PDW, Platelet Distribution Width; Pct, Plateletcrit; ESR, Erythrocyte Sedimentation Rate.

* Significant Value (p<0.05).

**Figure 1.**
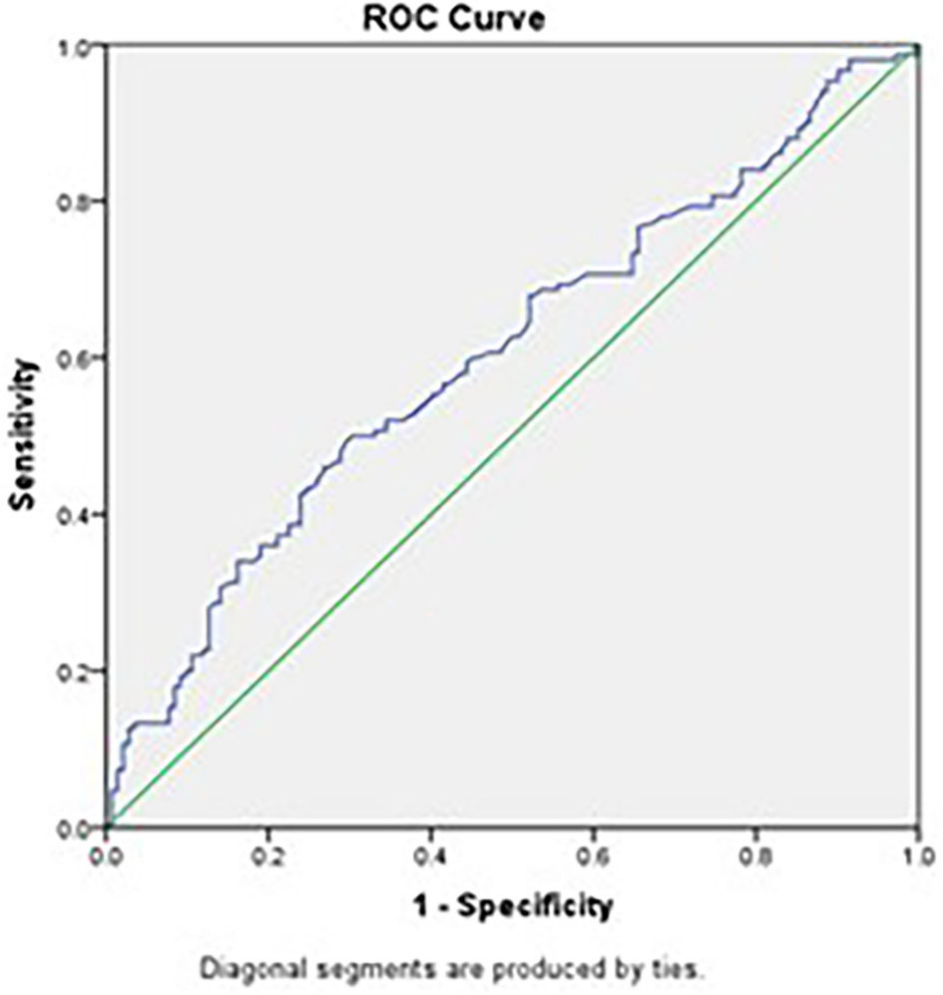
ROC curve depicting Platelet Count as a tool for differentiating Pulmonary. Tuberculosis from Pneumonia (Source: Şahin F, et al., 2012). ^3^

ROC curve analysis was done for Platelet count, Pct and ESR among Tuberculosis patients versus Pneumonia patients. The ROC analysis showed a Platelet count cutoff value 312,500/μl for differentiating Tuberculosis from Pneumonia. This cutoff provides a sensitivity of 62.7%, specificity of 50.0%, Positive Predictive Value of 57.0% and Negative Predictive Value of 55.9% ( Figure 1).

**Figure 2.**
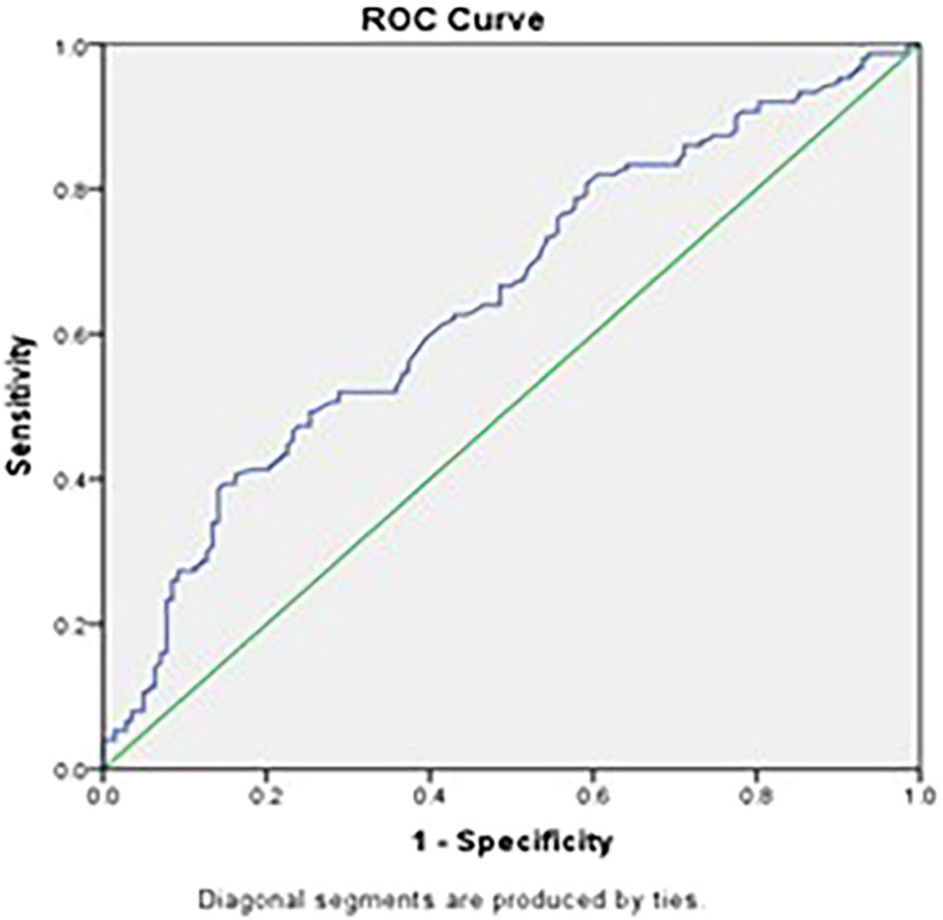
ROC curve using Pct in differential diagnosis of Pulmonary Tuberculosis and Pneumonia. (Source: Şahin F, et al., 2012). ^3^

Similarly, a Pct cutoff was also calculated to differentiate between Tuberculosis and Pneumonia. This value, defined to be 0.21%, provides a slightly higher sensitivity of 66.7%, specificity of 51.4%, Positive Predictive Value of 59.18% and Negative Predictive Value of 59.37% compared to that of the Platelet count cutoff ( Figure 2).

**Figure 3.**
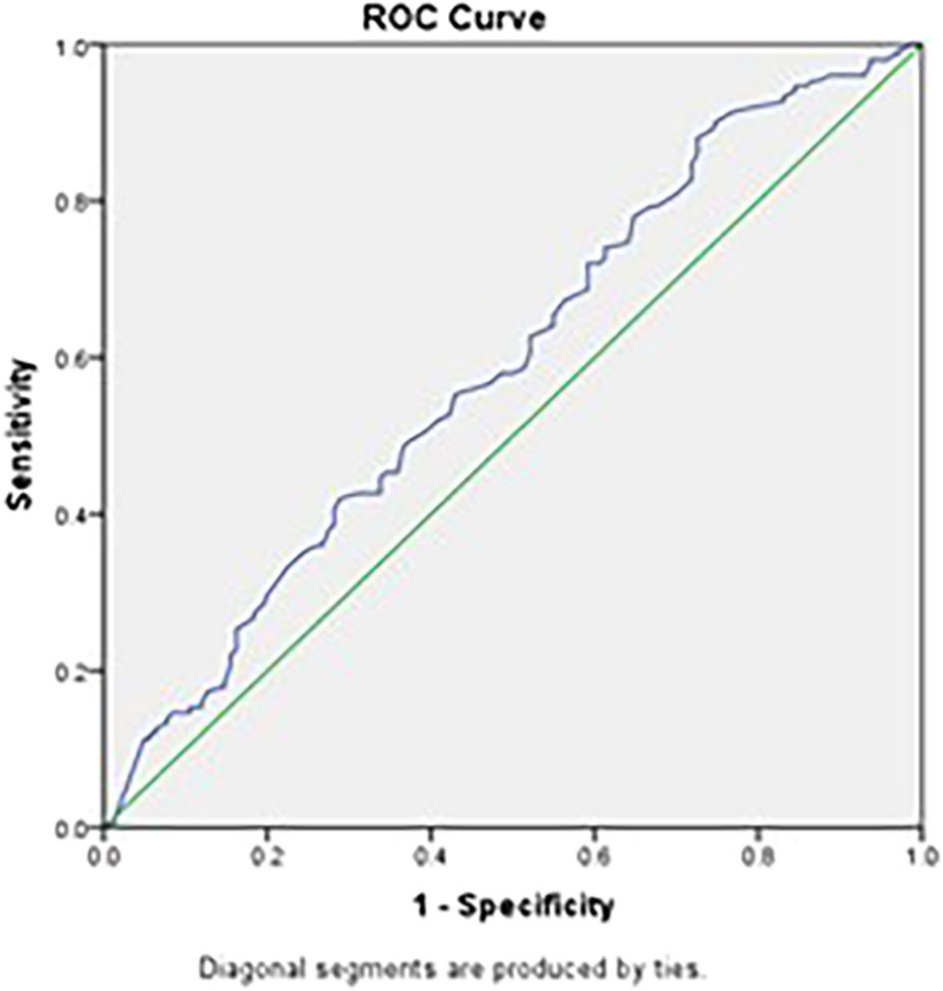
ROC curve using ESR to differentiate between Pulmonary Tuberculosis and Pneumonia. (Source: Şahin F, et al., 2012). ^3^

An ESR cutoff of 66.5 mm/hour for differentiating between Tuberculosis and Pneumonia provides a sensitivity of 58.0%, specificity of 51.4%, Positive Predictive Value of 55.76% and Negative Predictive Value of 53.67% ( Figure 3).

## Discussion

Platelets have long been recognized for their critical role in hemostasis. However, recent research has highlighted their significant involvement in inflammatory and infectious diseases. ^10, 11, 14, 20, 21^ This study supports previous findings by Sahin et al. and Tozkoparan et al., demonstrating that platelet counts are significantly elevated in patients with pulmonary tuberculosis (PTB) and pneumonia compared to healthy controls. ^3, 5^

The thrombocytosis observed in PTB patients may be due to the increased levels of inflammatory cytokines such as IL-1 and IL-6, which stimulate platelet production in the bone marrow. ^10, 11^ Elevated platelet counts in both PTB and pneumonia can be attributed to the action of these inflammatory interleukins produced during the immune response. Specifically, IL-1 and IL-6 promote bone marrow cells to enhance platelet synthesis, resulting in thrombocytosis. ^10^ Additionally, pro-inflammatory mediators like IL-6 can induce megakaryocytes to release larger platelets, initially increasing the mean platelet volume (MPV). As inflammation progresses, these larger platelets are recruited to sites of inflammation, leading to a subsequent decrease in circulating MPV. ^22– 24^

The trends in MPV across different studies have been inconsistent. Some studies report an increase in MPV during acute inflammatory responses due to the release of larger platelets, ^8– 10, 15^ while others observe a decrease in MPV as these larger platelets migrate to inflammation sites. ^22, 24^ In our study, MPV was significantly lower in both PTB and pneumonia groups compared to controls. This suggests that the migration of larger platelets to areas of inflammation may be the predominant process in these conditions.

Furthermore, our findings indicate that both platelet distribution width (PDW) and plateletcrit (Pct) were elevated in PTB patients. This is consistent with previous research, which shows that these indices reflect thrombocytosis and platelet activation. ^3, 4^ An elevated PDW indicates greater variability in platelet size, likely in response to the varied functional demands during inflammation. Similarly, an increased Pct, which represents the volume percentage of platelets in the blood, suggests a higher platelet mass as part of the inflammatory response.

There is a positive correlation between platelet counts, Pct, and erythrocyte sedimentation rate (ESR), highlighting the interconnectedness of these markers in indicating the inflammatory state. ESR, a well-established marker of inflammation, correlates with platelet indices, further supporting the role of platelets in the inflammatory process. ^3, 5^

Receiver Operating Characteristic (ROC) curve analysis in our study revealed that platelet count and Pct have moderate diagnostic value in distinguishing PTB from pneumonia, with areas under the curve (AUC) of 0.604 and 0.648, respectively. Although the sensitivity and specificity are limited, these parameters can be valuable components of a comprehensive diagnostic approach. Combining platelet indices with other clinical and laboratory findings may improve diagnostic accuracy. ^3^ Our results are consistent with those of Sahin et al. and Tozkoparan et al., who also reported elevated platelet counts in PTB compared to pneumonia and control groups. ^3, 5^ However, discrepancies in MPV trends across studies may result from differences in study populations, stages of inflammation, and methodological approaches. ^12, 14, 15^ Understanding these variations is essential for accurately interpreting platelet dynamics in inflammatory diseases.

The limitation of the study is that it did not assess changes in platelet indices over the course of treatment, which could provide insights into their prognostic value. This limitation can be overcome by conducting prospective studies with larger and more diverse populations are needed to validate the diagnostic utility of platelet indices. Additionally, investigating the underlying mechanisms of platelet activation in PTB and pneumonia could provide deeper insights into their role in inflammation. Exploring the integration of platelet indices with other biomarkers may enhance diagnostic accuracy and clinical decision-making.

## Conclusion

Platelet counts and indices are significantly altered in patients with pulmonary tuberculosis and pneumonia and show strong correlations with ESR levels. These findings highlight the role of platelets as key inflammatory mediators in respiratory infections. The distinct patterns of platelet parameter changes suggest their potential utility as diagnostic and prognostic markers, extending our understanding of platelets beyond their traditional hemostatic functions to their involvement in the immune response.

## Author contributions

Dr. Seemitr Verma conceptualized the study, developed the methodology, and supervised the project, as well as analyzed and interpreted the data and prepared the manuscript. Dr. Ruchee Khanna provided expertise in pathology, contributed to the study design, and reviewed the manuscript for critical intellectual content. Dr. Vinay Khanna assisted in study design and methodology, performed laboratory experiments, and contributed to the interpretation of results. Saisreeram Kandula conducted data collection, participated in data analysis, and helped draft sections of the manuscript. Vikram Ram Rajgopal assisted with laboratory experiments and data collection and participated in discussions regarding the results. Vishnu Prasad contributed to data collection and analysis and provided input during the manuscript writing process.

## Ethics and consent

Ethical approval for this study was obtained from the institutional ethics committee (IEC no. 673/2019) of Kasturba hospital, Manipal on 28 ^th^ January 2020. All procedures were conducted in accordance with the ethical principles outlined in the Declaration of Helsinki ( https://www.wma.net/policies-post/wma-declaration-of-helsinki-ethical-principles-for-medical-research-involving-human-subjects/). Patient data were anonymized to protect privacy and confidentiality was rigorously maintained throughout the study, in line with the established ethical guidelines. Since this is a retrospective study, the ethical committee has waived off the consent.

## Data Availability

Figshare: *Platelet-Based Biomarkers: A New Frontier in Inflammation Monitoring for Respiratory Infections.* Doi: https://doi.org/10.6084/m9.figshare.28218782.v1. ^25^ This project contains the following underlying data:
• pneumonia final.xlsx • tuberculosis final.xlsx • control 2 final.xlsx Data are available under the terms of the Creative Commons Attribution 4.0 International license (CC-BY 4.0).
