# Supplementary material for: Platelet-based biomarkers: A new frontier in inflammation monitoring for respiratory infections
Source: F1000Res. 2025 Mar 25;14:328. [Version 1] doi: 10.12688/f1000research.161124.1 (PMC12961282; doi:10.12688/f1000research.161124.1)
Supplement: Peer review history

# Reviewer report: Reviewer response for version 1

**DOI:** 10.5256/f1000research.177116.r455863

**Published:** 2026-03-04

**Authors:** Sonal Jain

**Affiliations:** 1 Heidelberg University, Heidelberg, Germany

**Summary**

 This manuscript titled “Platelet-based biomarkers: A new frontier in inflammation monitoring for respiratory infections” presents a retrospective case control study evaluating platelet indices as potential biomarkers for inflammation and diagnostic differentiation in pulmonary tuberculosis (PTB) and pneumonia. The study includes 450 participants and demonstrates statistically significant differences in platelet parameters between disease groups and healthy controls, with moderate correlations with ESR.

 While the findings support an association between platelet indices and inflammatory status, the reported diagnostic performance is modest (sensitivity and specificity ~50–67%), indicating limited utility as standalone diagnostic markers. The authors appropriately suggest these indices may be useful as adjunctive markers. While the study contributes useful data on platelet indices in respiratory infections, some methodological and reporting clarifications can be made to strengthen the overall quality of the manuscript.

**Major comments**

1. It is unclear whether patients were treatment-naive or receiving therapy at the time of blood collection. Please clarify treatment status and timing of sample collection.
2. The use of healthy controls may overestimate diagnostic performance. In clinical settings, differentiation from other symptomatic respiratory conditions is more relevant. This should be acknowledged as a limitation.
3. Please include a summary table of demographic and clinical characteristics of the participants.
4. The rationale for focusing specifically on PTB and pneumonia should be clarified, including whether platelet indices may distinguish these from other respiratory infections.

**Minor comments**

1. Clarify diagnostic criteria used for PTB and pneumonia, with reference to established guidelines.
2. Improve figure resolution, as text is difficult to read.
3. Ensure consistent use of abbreviations and capitalization.
4. Minor grammatical and typographical errors should be corrected.

Is the work clearly and accurately presented and does it cite the current literature?

Yes

If applicable, is the statistical analysis and its interpretation appropriate?

Yes

Are all the source data underlying the results available to ensure full reproducibility?

Yes

Is the study design appropriate and is the work technically sound?

Yes

Are the conclusions drawn adequately supported by the results?

Yes

Are sufficient details of methods and analysis provided to allow replication by others?

Yes

Reviewer Expertise:

Molecular diagnostics, Tuberculosis, Molecular biology, bioengineering, infectious diseases

I confirm that I have read this submission and believe that I have an appropriate level of expertise to confirm that it is of an acceptable scientific standard.

# Reviewer report: Reviewer response for version 1

**DOI:** 10.5256/f1000research.177116.r376191

**Published:** 2025-05-26

**Authors:** Stephine g

**Affiliations:** 1 university of toronto, toronto, USA

This article investigates the potential of platelet parameters as biomarkers for monitoring inflammation in respiratory infections, specifically comparing pulmonary tuberculosis (PTB) and pneumonia with healthy controls. The study analyzed platelet count, mean platelet volume (MPV), plateletcrit (PCT), platelet distribution width (PDW), and erythrocyte sedimentation rate (ESR) in 450 participants (158 PTB patients, 142 pneumonia patients, and 150 healthy controls). The findings suggest that platelet indices correlate with inflammatory markers and may serve as non-invasive diagnostic tools for respiratory infections. The research addresses an important and emerging area in diagnostic medicine.

 - The sample size is adequate for this type of study.

 - The statistical analysis is generally appropriate for the study objectives.

 - The presentation of ROC curve analyses provides useful information on potential diagnostic cutoff values.

 - The study explores correlations between multiple platelet parameters and established inflammatory markers.

 This manuscript addresses an important topic and presents potentially valuable findings regarding platelet parameters in respiratory infections. However,  conclusions should be tempered to reflect the moderate diagnostic performance demonstrated by the results.

Is the work clearly and accurately presented and does it cite the current literature?

Yes

If applicable, is the statistical analysis and its interpretation appropriate?

Yes

Are all the source data underlying the results available to ensure full reproducibility?

Yes

Is the study design appropriate and is the work technically sound?

Yes

Are the conclusions drawn adequately supported by the results?

Yes

Are sufficient details of methods and analysis provided to allow replication by others?

Yes

Reviewer Expertise:

Haematology,cancer biology

I confirm that I have read this submission and believe that I have an appropriate level of expertise to confirm that it is of an acceptable scientific standard.
